# Supplementary material for: Neurexins in serotonergic neurons regulate neuronal survival, serotonin transmission, and complex mouse behaviors
Source: eLife. 2023 Jan 25;12:e85058. doi: 10.7554/eLife.85058 (PMC9876567; doi:10.7554/eLife.85058)

bAct_Brainstem_unedited blot

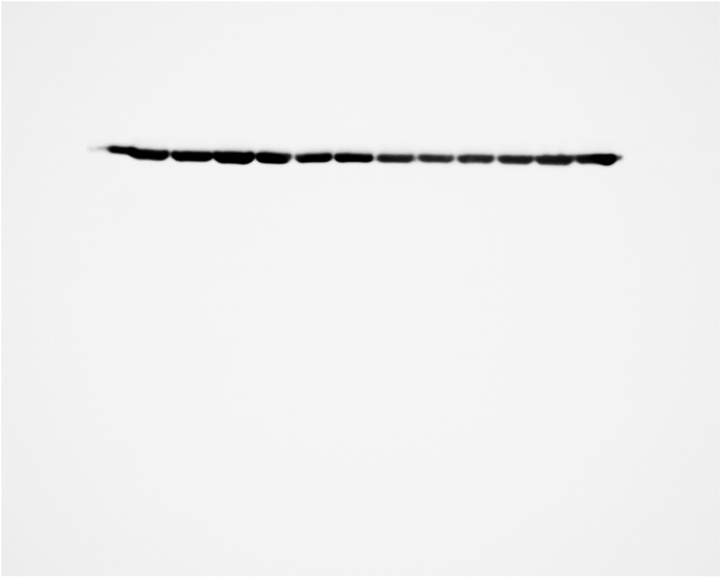

bAct_Hip_unedited blot

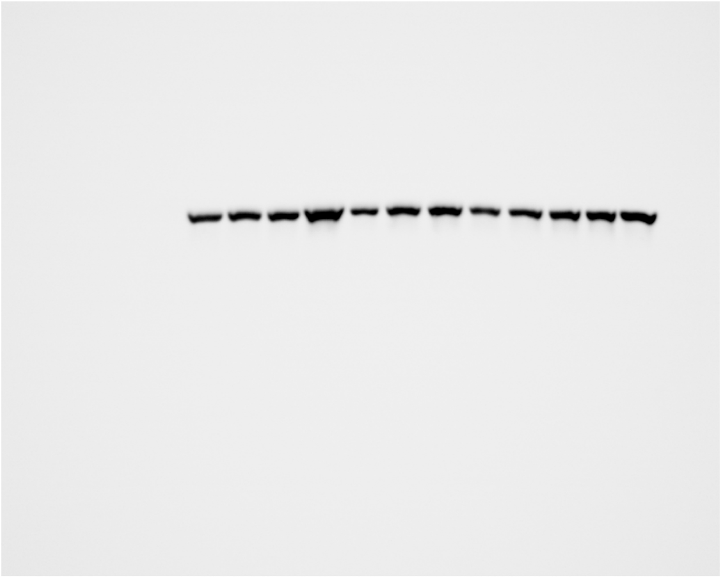

Sert_Brainstem_unedited blot

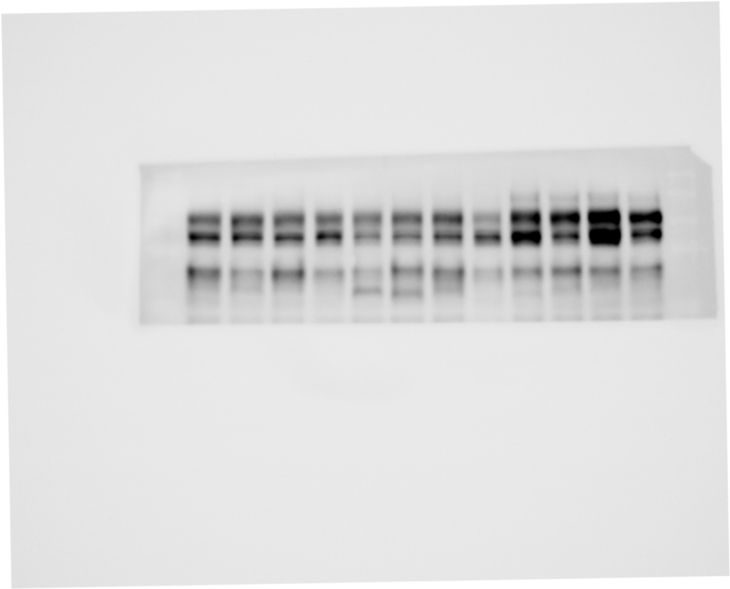

Sert_Hip_unedited blot

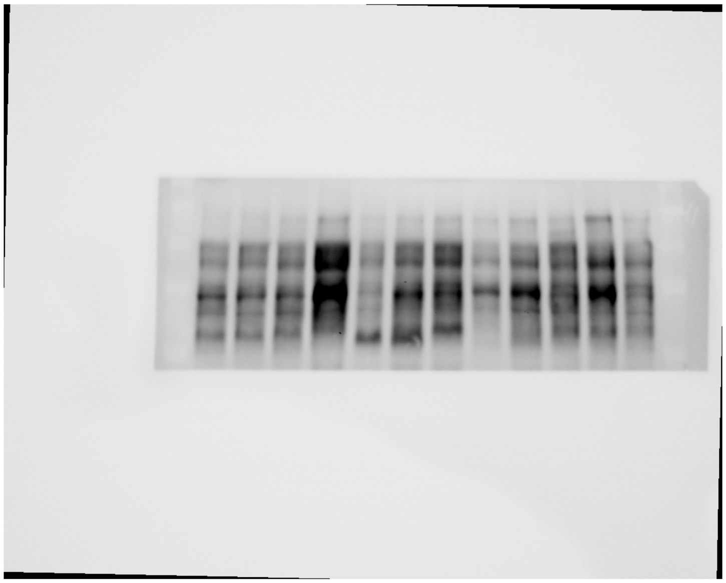

Supplement: Figure 3—source data 1. [file elife-85058-fig3-data1.zip › Figure 3 Figure Supplement 1 Source Data File 2.pdf]
