# Supplementary figures and images for: Neurexins in serotonergic neurons regulate neuronal survival, serotonin transmission, and complex mouse behaviors
Source: eLife. 2023 Jan 25;12:e85058. doi: 10.7554/eLife.85058 (PMC9876567; doi:10.7554/eLife.85058)

## Brainstem

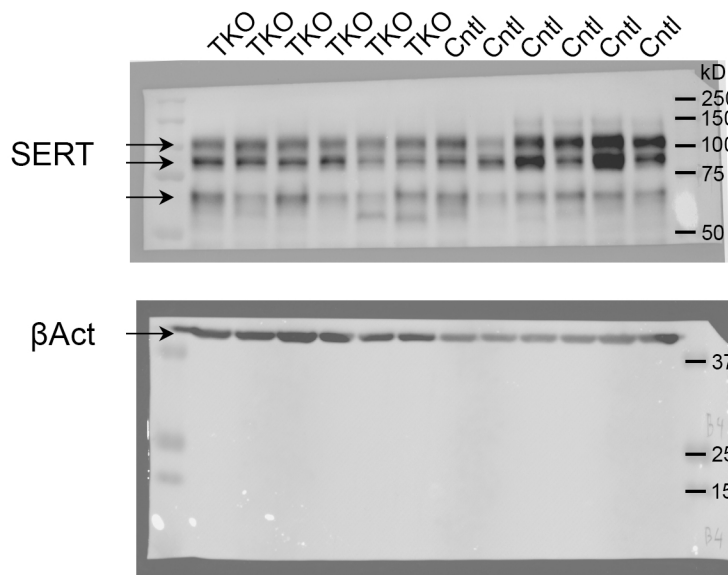

## Hippocampus

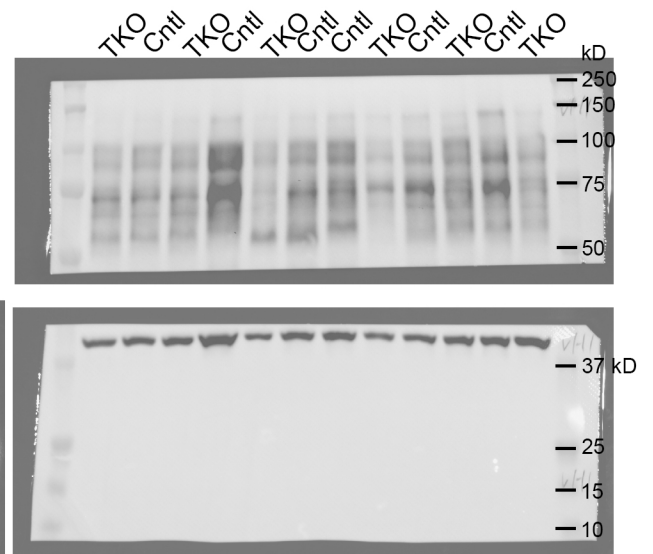

Supplement: Figure 3—source data 1. [file elife-85058-fig3-data1.zip › Figure 3 Figure Supplement 1 Source Data File 1.pdf]
